# A Secure and Fair Joint E-Lottery Protocol

**DOI:** 10.1155/2014/139435

**Published:** 2014-05-04

**Authors:** Chin-Ling Chen, Yuan-Hao Liao, Woei-Jiunn Tsaur

**Affiliations:** ^1^Department of Computer Science and Information Engineering, Chaoyang University of Technology, Taichung 41349, Taiwan; ^2^Department of Information Management, Da-Yeh University, Changhua 51591, Taiwan

## Abstract

The attractive huge prize causes people to adore lotteries. Due to the very small probability of winning prizes, the players can enhance their probability of winning by using the method of joint purchase. In spite of many lottery schemes having been proposed, most e-lottery schemes focus on the players' privacy or computation overhead rather than support a joint purchase protocol on the Internet. In this paper, we use the multisignature and verifiable random function to construct a secure and fair joint e-lottery scheme. The players can check the lottery integrity, and the winning numbers can be verified publicly.

## 1. Introduction

Gambling has the property of nonpredictability and attractive prizes. Players have the chance to obtain a huge prize but of course they cannot predict who the winner will be. Hence, gambling is very fascinating for many people, and the lottery is one kind of popular gambling [1–3]. The players must select their favorite numbers and pay money to purchase lottery tickets. After the deadline of the purchasing phase, the lottery organization (LO) randomly generates the winning numbers. If no one wins the lottery, the prize money will accumulate for the next round. The attractive huge prizes are an extremely powerful factor causing people to purchase lottery tickets and the main reason remains popular among players.

In past years, many lottery schemes were proposed. In 2006, Chow et al. [4] proposed practical electronic lotteries with an offline trusted third party (TTP); their scheme can satisfy all of the identified requirements without the presence of TTP for generating the winning numbers; the result of this generation is publicly verifiable.

Next, Lee and Chang [5] proposed an electronic *t*-out-of-*n* lottery on the Internet in 2009. The scheme is based on the Chinese Remainder Theorem that allows lottery players to simultaneously select *t* out of *n* numbers in a ticket without iterative selection. The drawback of this scheme is that the computation overhead of players in purchasing lotteries is too heavy.

In the same year, Lee et al. [6] proposed noniterative privacy preservation for online lotteries. This scheme not only allows players to choose *t*-out-of-*n* numbers in lotteries without iterative selection but also preserves the privacy of players' choices. Nevertheless, the computation overhead in purchasing lotteries is still heavy for the player who accesses the Internet with mobile or wireless devices.

In an overview of the above schemes, we find that the majority of the schemes focus on the players' privacy or computation overhead but cannot support a joint purchase protocol on the Internet.

Due to the probability of winning prize being very small [7], the players can employ two strategies of purchase to enhance the probability of winning prizes as follows.

- The player invites other players to collect more cash, and then the player purchases the sequential numbers to increase the probability of obtaining a prize.
- The player pays a small amount of money to purchase the lotteries in cooperation with other players.

To the best of our knowledge, there exist only two websites, called “e-Lottery Syndicates” [8] and “Myleto” [9], which provide a trading platform (TP) for purchases and a proxy purchase service. The difference between the above websites is that the former provides individual purchases while the latter provides joint purchases. Since our scheme focuses on joint purchases, we chose “Myleto” to discuss joint purchases.

The process for joint purchase in “Myleto” enables the players to bet their favorite numbers by using the “Myleto,” and then “Myleto” counts the preferred numbers of players to generate the popular numbers after the deadline of purchase phase. Then, “Myleto” takes the popular numbers to purchase the lottery for the trusted lottery organization (LO). Finally, LO generates the winning numbers and publishes them on the bulletin board. Then “Myleto” distributes the different prizes to the winning players according to the numbers they bet.

Even if the solution for the joint purchase lottery exists, according to our observations some drawbacks remain.

1. From the user's viewpoint, the risks are as follows:
  - if the joint purchase players win the first prize, the person receiving the award has a chance to abscond with the funds;
  - the player's lottery purchase evidence depends on the picture at the time of purchase and the credit card transaction receipt. However, the former lacks credibility because it is easy to fake, and the latter lacks immediacy since the credit card transaction receipt adopts a monthly settlement;
  - if the player's purchase information is lost or the TP refuses to give out the prize, the player cannot proffer strong evidence to prove the winner is himself/herself.
2. From the TP's viewpoint, the risk is as follows:
  - if a malicious player forges a picture and a credit card transaction receipt to claim the prize, the TP will find it hard to recognize whether the prize claim evidence is true or false.

At present, we have seen that the current TP of joint purchase exhibits some drawbacks, so determining how to implement a fair and secure joint purchase e-lottery protocol is still an open issue.

Hence, we propose a fair and secure joint e-lottery protocol to guarantee the rights and interests of the players and TP. Simultaneously, our proposed protocol also supports individual purchases.

The proposed scheme must be able to achieve the following requirements [4–6] such that the proposed scheme can be applied in actual practice.

1. *Public Verification*. All the valid lottery tickets and the winning numbers must be verified via a verifiable random function.
2. *Fairness*. No one can predict the winning result before the winning numbers are published.
3. *Security*. No one can forge a winning lottery or impersonate a lottery winner to claim the prize.
4. *Correctness*. The players can verify the public information of the bulletin board by themselves.
5. *Anonymity*. Including lottery agents, no one can identify the participants by the lottery ticket.
6. *Convenience*. The legitimate players should be able to purchase lottery via Internet.
7. *Without Preregistration*. Players need not register at any lottery agent or drawing center in advance, as registration in advance is unnecessary; this requirement should conform to an electronic lottery to make it more realistic.
8. *No Online Trusted Third Party (TTP)*. An electronic lottery is said to be impractical if the security of the entire mechanism depends on an online trusted third party.
9. *Participants' Legality*. The scenario of the joint e-lottery scheme should ensure the participants' legality via a multisignature.
10. *Support Joint and Individual E-Lottery Service*. The protocol must support joint and individual e-lottery service, respectively.

The remainder of this paper is organized to describe and analyze our joint e-lottery scheme as follows.  Section 2 introduces related cryptographic techniques used in our scheme.  Section 3 presents our proposed protocol, and the security requirements are analyzed in Section 4. Our conclusions are presented in the final section.

## 2. Preliminaries

In this section, we introduce three cryptographic techniques used in our scheme: a verifiable random function, an identity-based signature scheme, and an efficient identity-based RSA multisignature scheme.

### 2.1. Verifiable Random Function

A verifiable random function (VRF) was first proposed by Micali et al. [10]. Essentially, it is a pseudorandom function [11] providing noninteractively verifiable proof of the output's correctness. Therefore, the above properties of VRF are suitable for our scheme.

On the basis of the notation in [12], a set of functions *F*_(·)_(·):{0,1}^*k*^ → {0,1}^*l*(*k*)^ is a verifiable function; suppose there exist polynomial-time algorithms Gen (·), Eval (·, ·), Prove (⋯), Verify (·, ·, ·, ·) such that

(1) Gen(*k*)  is a probabilistic algorithm to generate a secret key SK that is generated by a random function and the corresponding public key PK that enables public verification;
(2) Eval(SK, *x*)  is an algorithm that computes the VRF's output *y* = *F*_SK_(*x*);
(3) Prove(SK, *x*) is an algorithm that computes the proof *π* that *y* = *F*_SK_(*x*);
(4) Verify(PK, *x*, *y*, *π*)  is an algorithm that verifies *y* = *F*_SK_(*x*);
(5) the VRF should satisfy the following properties.
(6) uniqueness:
  (1) $$\begin{array}{c} \text{Verify}\;\left(\text{PK},x,y_{1},\pi_{1}\right)=\text{Verify}\;\left(\text{PK},x,y_{2},\pi_{2}\right), \end{array}$$
  where *y*_1_ = *y*_2_;
(7) computability: Eval(SK, *x*) = *F*_SK_(*x*) is efficiently computable;
(8) provability: (*y*, *π*) = (Eval(SK, *x*), Prove (SK, *x*)) and Verify(PK, *x*, *y*, *π*);
(9) pseudorandomness: the probability that an attacker can input any bit of *F*_SK_(*x*) for *x* his/her choice is negligible even if she/he has seen the values of many *F*_SK_(*x*′) given *x*′ ≠ *x*.

### 2.2. Review of Shamir's Identity-Based Signature Scheme

In 1985, in order to simplify the public key authentication problem, Shamir [13] first offered the concept of an identity-based (ID-based) cryptosystem. In this system, each signer needs to register with a private key generator (PKG) and identify himself/herself before accessing the network resource. Once the registration is completed, the PKG will use the signer's identity to generate the secret key. The signer's identity may include the signer's name, email, and address. The advantage of this scheme is that there is no need for a public key directory in the system. The communicating parties only need to know the “identity” of his/her communication partner and the public key of the PKG is able to verify the signature or send an encrypted message.

We first introduce the notations used to explain how Shamir's scheme was constructed:

 (*p*_*X*_, *q*_*X*_): a pair of large prime numbers;
 
*N*_*X*_: a large number, where *N*_*X*_ = *p*_*X*_ · *q*_*X*_, *φ*(*N*_*X*_) = (*p*_*X*_ − 1)(*q*_*X*_ − 1), and *φ*(·) is Euler's totient function;
 (*e*_*X*_, *d*_*X*_): *X*'s public and private key, respectively, where *e*_*X*_ *d*_*X*_ = 1mod⁡*φ*(*N*_*X*_);
 
*H*(·): a one way hash function;
 
*m*: a message;
 
$A\underline{\underline{?}}B$: comparing whether or not *A* is equal to *B*.

#### 2.2.1. Private Key Generator (PKG) Keys

The private key generator (PKG) chooses its public and private key pair as follows.

**Step 1**

Run the probabilistic polynomial algorithm *K*_RSA_ to generate two random large primes, *p*_PKG_ and *q*_PKG_.

**Step 2**

Choose a random public key *e*_PKG_ such that *gcd*(*e*_PKG_, *ϕ*(*N*_PKG_)) = 1 and compute the private key *d*_PKG_ = *e*_PKG_^−1^mod⁡*ϕ*(*N*_PKG_).

#### 2.2.2. Signer Secret Key Generation

In this algorithm, the signer gets a copy of his/her secret key from the PKG through a two-step process.

**Step 1**

A signer submits his/her identity to the PKG.

**Step 2**

The PKG uses its private key *d*_PKG_ to sign the signer's identity *i* by generating the secret key *g* such that *g* = *i* ^*d*_PKG_^ mod⁡ *N*_PKG_.

#### 2.2.3. Message Signing

To sign a message *m*, the signer with the secret key *g* and the corresponding public key *e*_PKG_ of the PKG signs a message *m* by generating a signature pair *σ* = (*T*, *S*) as follows.

**Step 1**

Select a random number *r* and compute

(2) $$\begin{array}{c} T=r^{e_{\text{PKG}}}\mathrm{mod}N_{\text{PKG}}. \end{array}$$

**Step 2**

For the same random number *r*, compute

(3) $$\begin{array}{c} S=g\cdot r^{H(T,m)}\mathrm{mod}N_{\text{PKG}}. \end{array}$$

*σ* = (*T*, *S*) is the complete signature of the message *m*.

#### 2.2.4. Message Verification

The identity-based signature *σ* = (*T*, *S*) of a signer with identity *i* is valid if and only if the following equality holds:

(4) $$\begin{array}{c} S^{e}=i\cdot T^{H(T,m)}\mathrm{mod}N_{\text{PKG}}. \end{array}$$

### 2.3. Review of Harn's Efficient Identity-Based RSA Multisignatures Scheme

In the 2008, Harn and Ren [14] first proposed a digital signature of a message generated by multiple signers with multiple private keys based on Shamir's identity-based signature (IBS) scheme. This was a first efficient identity-based RSA multisignatures scheme with both fixed length and verification time. Harn and Ren's scheme is secure against forgeries under chosen-message attack, against multisigner collusion attack, and adaptive chosen-identity attack.

#### 2.3.1. Private Key Generator (PKG) Keys

The PKG chooses its public and private key pairs as follows.

**Step 1**

Runs the probabilistic polynomial algorithm *K*_RSA_ to generate two random large primes, *p*_PKG_ and *q*_PKG_.

**Step 2**

Choose a random public key *e*_PKG_ such that *gcd*(*e*_PKG_, *ϕ*(*N*_PKG_)) = 1 and compute the private key *d*_PKG_ = *e*_PKG_^−1^mod⁡*ϕ*(*N*_PKG_).

#### 2.3.2. Signer Secret Key Generation

In this algorithm, the signer gets a copy of his/her secret key from the PKG through a two-step process.

**Step 1**

A signer submits his/her identity to the PKG.

**Step 2**

The PKG uses its private key *d*_PKG_ to sign the message digest of the identity to generate the secret key *g*_*j*_, such that *g*_*j*_ = *i*_*j*_^*d*_PKG_^mod⁡*N*_PKG_. No one will be able to distinguish between the identity and its message digest *i*_*j*_.

#### 2.3.3. Message Signing

To generate an identity-based multisignature, each signer carries out the followings steps.

**Step 1**

Choose a random integer *r*_*j*_ and compute *t*_*j*_ = *r*_*j*_^*e*_PKG_^mod⁡*N*_PKG_.

**Step 2**

Broadcast *t*_*j*_ to other signers.

**Step 3**

Upon receiving of *t*_*j*_, *j* = 1,2,…, *l*. each signer computes

(5) $$\begin{array}{c} T=\overset{l}{\underset{j=1}{\prod }}t_{j}\;\mathrm{mod}N_{\text{PKG}}\\ s_{j}=g_{j}\cdot r_{j}^{H(T,m)}\mathrm{mod}N_{\text{PKG}}. \end{array}$$

**Step 4**

Broadcast *s*_*j*_ to all signers.

**Step 5**

After receiving of *t*_*j*_, *j* = 1,2,…, *l*. the multisignatures component *S* can be computed as

(6) $$\begin{array}{c} S=\overset{l}{\underset{j=1}{\prod }}s_{j}\;\mathrm{mod}N_{\text{PKG}}. \end{array}$$

The multisignature for message *m* is *σ* = (*T*, *S*).

#### 2.3.4. Multisignature Verification

To verify a multisignature *σ* = (*T*, *S*) of a message *m* of signers whose identities are *i*_1_, *i*_2_,…, *i*_*l*_, anyone can verify the correctness as follows:

(7) $$\begin{array}{c} S^{e_{\text{PKG}}}\;\underline{\underline{?}}\;\left(i_{1},i_{2},\ldots ,i_{l}\right)\cdot T^{H(T,m)}\mathrm{mod}N_{\text{PKG}}. \end{array}$$

## 3. The Proposed Joint E-Lottery Protocol

**Figure 1:**
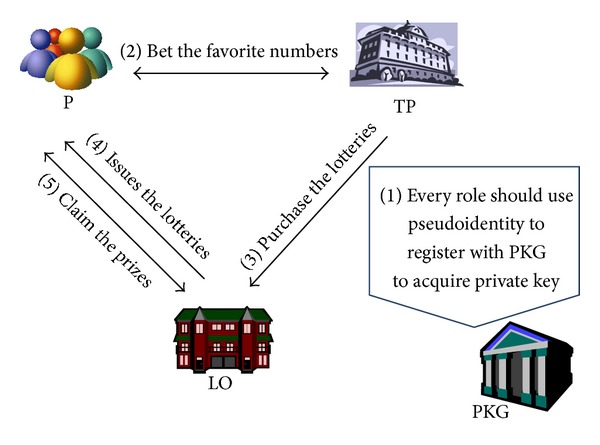
The structure of our scheme.

The structure of our scheme is illustrated in Figure 1.

There are four participants involved in the proposed e-lottery scheme.

1. *Private Key Generator (PKG)*. The off-line trusted third party which generates private keys to all participants.
2. *Player (P)*. The player is a participator in the lottery gamble.
3. Trading* Platform (TP)*. The trading platform is a website to provide players for joining the e-lottery game.
4. *Lottery Originator (LO)*. The LO issues the lotteries, generates the winning numbers to sell lotteries to gain revenue, and gives out the prizes.

**Step 1**

P, TP, LO ↔ PKG: all participants must register to PKG to acquire their private key with his/her pseudoidentity.

**Step 2**

P ↔ TP: the players bet their favorite numbers to the TP.

**Step 3**

TP→LO: the TP gathers the statistics on the betting numbers to generate the majority of popular numbers and then purchases the popular numbers with the LO.

**Step 4**

LO→P: the LO issues lotteries to the players.

**Step 5**

P ↔ LO: after the winning numbers are announced, the winning players use their winning lotteries and private keys to claim the prizes won.

The following notations are used in our protocol:

 number_*j*_: the favorite numbers of the *j*th player;
 chain_*j*_: the published hash chain set of the valid random seed *β*_*j*_ generated by player, which is involved in generating the winning number, where chain_0_ is the initial vector; the chain_0_ = 0, chain_1_ = *H*(chain_0_, *β*_1_), chain_2_ = *H*(chain_1_, *β*_2_), and chain_*j*_ = *H*(chain_*j*−1_, *β*_*j*_);
 
*C*_*i*_: the *i*th ciphertext;
 
*σ*_*m*_: the identity-based signature of message *m*;
 
*m*_req_: the request message;
 
*i*_*j*_: the message digest of the *j*th player's identity;
 PL: the purchased list, where PL = (*i*_*j*_, *β*_*j*_);
 
*H*(·): one way hash function [15];
 
*r*_*X*_: the random number is selected by *X*;
 
*β*_*j*_: the hash value, where *β*_*j*_ = *H*(*r*_*X*_);
 
*K*_*X*-*Y*_: the session key between the *X* and *Y* which is constructed by IETF [16, 17];
 
*E*(*K*_*X*-*Y*_, (*m*)): an encryption function which uses the session key *K*_*X*-*Y*_ to encrypt the message *m*;
 
*D*(*K*_*X*-*Y*_, (*C*)): a decryption function which uses the session key *K*_*X*-*Y*_ to decrypt the ciphertext *C*.

### 3.1. Constructing the Session Key Model

Diffie and Hellman proposed a key agreement protocol [18] in 1978. The RFC 2631 was drawn up for the key agreement protocol in 1999 by the IETF. Therefore, we use the RFC 2631 protocol to construct the session keys. The session keys are used in our protocol with three situations. First, when the purchase is individual, the player must share the session key to protect his/her favorite numbers. Second, the TP and LO are jointed to sign the multisignature; they must share a common secret key to encrypt the signature. Third, the LO issues the lotteries to players, and the winning players send the claim prize message to the LO; they must also share a session key to encrypt or decrypt the messages.

### 3.2. The Initialization Phase

In this phase, the PKG performs the keys generating function to generate the public and private keys. On the other hand, the LO performs the VRF to generate the related functions and then publishes it.

**Step 1**

The PKG selects a random number *k* and then performs Gen(*k*) to generate the public *e*_PKG_ and private *d*_PKG_.

**Step 2**

The LO performs the VRF to generate the related functions that include Eval(·, ·), Prove(·, ·), and Verify(·, ·, ·).

### 3.3. The Registration Phase

In this phase, all of the roles submit their identities to the PKG to become legal participants. Notably, the players must submit their identities (including the players' name, email, and addresses) and a random number to PKG and then PKG signs the message digest of the identity by its *d*_PKG_ and *d*_PKG_.

The PKG computes the participants' private keys with its *d*_PKG_ as in the following equations:

(8) $$\begin{array}{c} d_{j}=i_{j}^{d_{\text{PKG}}}\mathrm{mod}N_{\text{PKG}}\;\text{for}\;j=1,2,\ldots ,n.\\ d_{\text{TP}}={\text{ID}}_{\text{TP}}^{d_{\text{PKG}}}\mathrm{mod}N_{\text{PKG}},\\ d_{\text{LO}}={\text{ID}}_{\text{LO}}^{d_{\text{PKG}}}\mathrm{mod}N_{\text{PKG}}. \end{array}$$

After that, the PKG publishes ID_TP_ and ID_LO_ on the bulletin board.

### 3.4. The Players Bet for Lottery Numbers Phase

In this phase, the players can bet their favorite numbers via the TP and then the TP publishes the purchase information on the bulletin board. When this phase is finished, the TP will send bulletin board information to the LO. According to the received information, the LO publishes the winning numbers. Moreover, players, TP, and LO can use the published bulletin board information to check whether or not the following three information items are correct.

1. The players' purchased lotteries are included in the hash chain.
2. The players' bet numbers are valid or not.
3. The players are legal or not.

The individual purchase is also included in the hash chain and the purchased information (including identity information, hash chain value, and hash value of the random number) is also published on the bulletin board, except for the selected favorite numbers.

**Figure 2:**
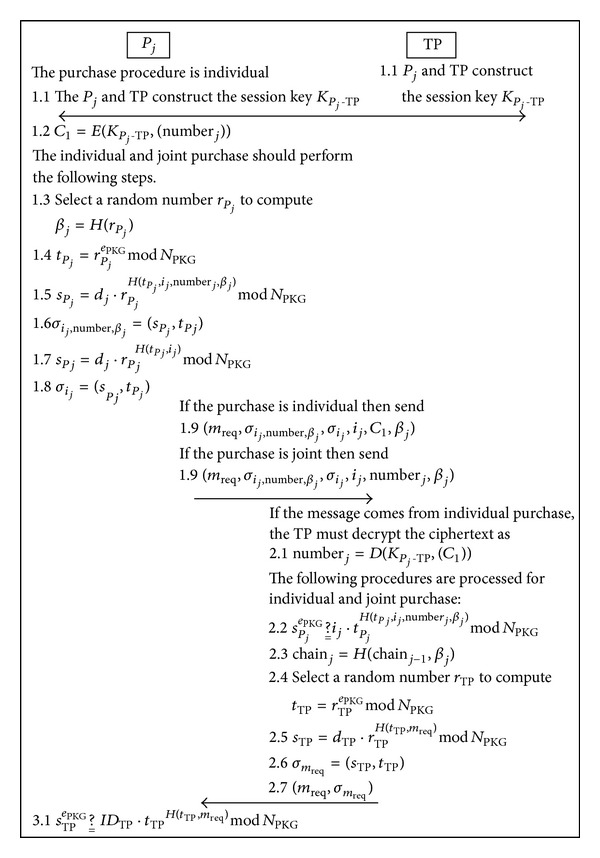
Overview of the players bet for lottery number phase.

The players bet for lottery numbers phase is illustrated in Figure 2, and the bulletin board information is illustrated in Table 1.

If anyone questions the players' legality then they can use the signature of the players' identity of the bulletin board to verify the legality of the players by

(9) $$\begin{array}{c} s_{P_{j}}^{e_{\text{PKG}}}\;\underline{\underline{?}}\;i_{j}\cdot t_{P_{j}}^{H(t_{P_{j}},i_{j})}\mathrm{mod}N_{\text{PKG}}. \end{array}$$

**Step 1**

If the purchase is individual then the player must compute session key *K*_*P*_*j*_-TP_ (refer to Section 3.1), using it to protect the individual's favorite number number_*j*_ as follows:

(10) $$\begin{array}{c} C_{1}=E\left(K_{P_{j}\text{-TP}},\left({\text{number}}_{j}\right)\right). \end{array}$$

The individual and joint purchases are both required to process (11)–(15).

Then, the *j*th player selects a random number *r*_*P*_*j*__to compute

(11) $$\begin{array}{c} \beta_{j}=H\left(r_{P_{j}}\right),\\ t_{P_{j}}=r_{P_{j}}^{e_{\text{PKG}}}\mathrm{mod}N_{\text{PKG}}. \end{array}$$

The *P*_*j*_ uses his/her private key *d*_*j*_ to compute

(12) $$\begin{array}{c} s_{P_{j}}=d_{j}\cdot r_{P_{j}}^{H(t_{P_{j}},i_{j},{\text{number}}_{j},\beta_{j})}\mathrm{mod}N_{\text{PKG}}. \end{array}$$

Here, we denote the signature as follows:

(13) $$\begin{array}{c} \sigma_{i_{j},{\text{number}}_{j},\beta_{j}}=\left(s_{P_{j}},t_{P_{j}}\right). \end{array}$$

Finally, the *P*_*j*_ uses his/her private key *d*_*j*_ to sign his/her identity *i*_*j*_ as follows:

(14) $$\begin{array}{c} s_{P_{j}}=d_{j}\cdot r_{P_{j}}^{H(t_{P_{j}},i_{j})}\mathrm{mod}N_{\text{PKG}}. \end{array}$$

We denote the signature as follows:

(15) $$\begin{array}{c} \sigma_{i_{j}}=\left(s_{P_{j}},t_{P_{j}}\right). \end{array}$$

The difference between the multisignature *σ*_*i*_*j*__ and *σ*_*i*_*j*_,number_*j*_,*β*_*j*__ is that the former is published on the bulletin board and all participants can use it to verify the player's legality, while the latter is used to achieve the message nonrepudiation for the TP.

Afterward, if the purchase is individual then the request message *m*_req_, signature *σ*_*i*_*j*_,number_*j*_,*β*_*j*__, and related parameters (*i*_*j*_, *C*_1_, *β*_*j*_) are sent to the TP.

If the purchase is joint, the request message *m*_req_, signature *σ*_*i*_*j*_,number_*j*_,*β*_*j*__, and related parameters (*i*_*j*_, number_*j*_, *β*_*j*_) are sent to the TP.

**Step 2**

After receiving the message, if it comes from individual purchase then the TP must decrypt the ciphertext *C*_1_ to obtain number_*j*_ and then the following procedures are processed for individual and joint purchases.

First, the TP checks the validity of signature as follows:

(16) $$\begin{array}{c} s_{P_{j}}^{e_{\text{PKG}}}\;\underline{\underline{?}}\;i_{j}\cdot t_{P_{j}}^{H(t_{P_{j}},i_{j},{\text{number}}_{j},\beta_{j})}\mathrm{mod}N_{\text{PKG}}. \end{array}$$

The TP links *β*_*j*_ into the hash chain as follows:

(17) $$\begin{array}{c} {\text{chain}}_{j}=H\left({\text{chain}}_{j-1},\beta_{j}\right). \end{array}$$

Next, the TP selects a random number *r*_TP_ to compute

(18) $$\begin{array}{c} t_{\text{TP}}=r_{\text{TP}}^{e_{\text{PKG}}}\mathrm{mod}N_{\text{PKG}}\\ s_{\text{TP}}=d_{\text{TP}}\cdot r_{\text{TP}}^{H(t_{\text{TP}},m_{\text{req}})}\mathrm{mod}N_{\text{PKG}}. \end{array}$$

Here, we denote the signature of *m*_req_ as follows:

(19) $$\begin{array}{c} \sigma_{m_{\text{req}}}=\left(s_{\text{TP}},t_{\text{TP}}\right). \end{array}$$

Finally, TP sends signature (*σ*_*m*_req__, *m*_req_) to the *P*_*j*_.

**Step 3**

After receiving (*σ*_*m*_req__, *m*_req_), *P*_*j*_ checks the validity of signature as follows:

(20) $$\begin{array}{c} s_{\text{TP}}^{e_{\text{PKG}}}\;\underline{\underline{?}}\;{\text{ID}}_{\text{TP}}\cdot t_{\text{TP}}^{H(t_{\text{TP}},m_{\text{req}})}\mathrm{mod}N_{\text{PKG}}. \end{array}$$

If the signature is invalid, then the *P*_*j*_ terminates the transaction.

### 3.5. The Purchase Phase

After the purchase deadline, the TP gathers the statistics on numbers to generate the popular numbers. Subsequently, the TP sends the purchase message that includes purchase list and the partial signature to the LO. Note that the lottery's numbers of individual purchase are determined by individual buyers rather than through counting by TP; individual purchase is the same as joint purchase.

1. The individual purchase is included in the purchase list.
2. The TP also computes the partial signature for individual purchase.

**Figure 3:**
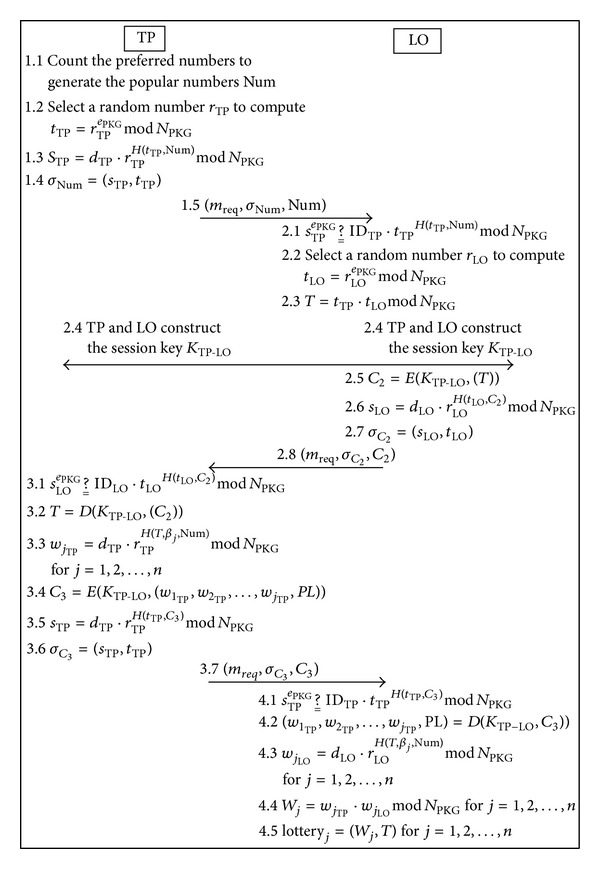
Overview of the purchase phase.

The overview of the purchase phase is illustrated in Figure 3.

**Step 1**

After the purchase deadline, the TP counts the preferred numbers of all of the players to generate the popular numbers Num; then the TP selects a random number *r*_TP_ to compute the partial signature *t*_TP_ and *s*_TP_ as follows:

(21) $$\begin{array}{c} t_{\text{TP}}=r_{\text{TP}}^{e_{\text{PKG}}}\mathrm{mod}N_{\text{PKG}}\\ s_{\text{TP}}=d_{\text{TP}}\cdot r_{\text{TP}}^{H(t_{\text{TP}},\text{Num})}\mathrm{mod}N_{\text{PKG}}. \end{array}$$

Here, we denote the signature of Num as follows:

(22) $$\begin{array}{c} \sigma_{\text{Num}}=\left(s_{\text{TP}},t_{\text{TP}}\right). \end{array}$$

Finally, the TP sends the request message *m*_req_, signature *σ*_Num_, and the popular numbers Num to the LO.

**Step 2**

Before receiving the message (*m*_req_, *σ*_Num_, Num), the LO checks the signature validity as follows:

(23) $$\begin{array}{c} s_{\text{TP}}^{e_{\text{PKG}}}\;\underline{\underline{?}}\;{\text{ID}}_{\text{TP}}\cdot t_{\text{TP}}^{H(t_{\text{TP}},\text{Num})}\mathrm{mod}N_{\text{PKG}}. \end{array}$$

Subsequently, the LO selects random number *r*_LO_ to compute

(24) $$\begin{array}{c} t_{\text{LO}}=r_{\text{LO}}^{e_{\text{PKG}}}\mathrm{mod}N_{\text{PKG}}. \end{array}$$

LO uses the partial signature of the TP and LO to compute *T* as follows:

(25) $$\begin{array}{c} T=t_{\text{TP}}\cdot t_{\text{LO}}\mathrm{mod}N_{\text{PKG}}. \end{array}$$

The TP and LO construct the session key *K*_TP-LO_ and then encrypt the partial signature as follows:

(26) $$\begin{array}{c} C_{2}=E\left(K_{\text{TP-LO}},\left(T\right)\right). \end{array}$$

The LO uses the private key *d*_LO_ to compute the partial signature of *C*_2_ as follows:

(27) $$\begin{array}{c} s_{\text{LO}}=d_{\text{LO}}\cdot r_{\text{LO}}^{H(t_{\text{LO}},C_{2})}\mathrm{mod}N_{\text{PKG}}. \end{array}$$

Here, we denote the signature of *C*_2_ as

(28) $$\begin{array}{c} \sigma_{C_{2}}=\left(s_{\text{LO}},t_{\text{LO}}\right). \end{array}$$

Finally, the LO sends (*m*_req_, *σ*_*C*_2__, *C*_2_) to TP.

**Step 3**

After receiving (*σ*_*C*_3__, *m*_req_), the TP checks the validity of signature as follows:

(29) $$\begin{array}{c} s_{\text{LO}}^{e_{\text{PKG}}}\;\underline{\underline{?}}\;{\text{ID}}_{\text{LO}}\cdot t_{\text{LO}}^{H(t_{\text{LO}},C_{2})}\mathrm{mod}N_{\text{PKG}}. \end{array}$$

The TP uses the session key *K*_TP-LO_ to decrypt the cipher text as follows:

(30) $$\begin{array}{c} T=D\left(K_{\text{TP-LO}},\left(C_{2}\right)\right). \end{array}$$

According to the purchased list PL, the TP uses its private key *d*_TP_ to compute the partial multisignatures *w*_*j*_TP__of the player's lottery as in (31) as follows:

(31) $$\begin{array}{c} w_{j_{\text{TP}}}=d_{\text{TP}}\cdot r_{\text{TP}}^{H(T,\beta_{j},\text{Num})}\mathrm{mod}N_{\text{PKG}},\;\text{for}\;j=1,2,\ldots ,n. \end{array}$$

To protect the message, the TP uses the session key *K*_TP-LO_ to encrypt parameters (*w*_1_TP__, *w*_2_TP__,…, *w*_*j*_TP__, PL) as follows:

(32) $$\begin{array}{c} C_{3}=E\left(K_{\text{TP-LO}},\left(w_{1_{\text{TP}}},w_{2_{\text{TP}}},\ldots ,w_{j_{\text{TP}}},\text{PL}\right)\right). \end{array}$$

The TP uses its private key *d*_TP_ to compute the partial signature of *C*_3_ as

(33) $$\begin{array}{c} s_{\text{TP}}=d_{\text{TP}}\cdot r_{\text{TP}}^{H(t_{\text{TP}},C_{3})}\mathrm{mod}N_{\text{PKG}}. \end{array}$$

Here, we denote the signature of *C*_3_ as follows:

(34) $$\begin{array}{c} \sigma_{C_{3}}=\left(s_{\text{TP}},t_{\text{TP}}\right). \end{array}$$

Afterward, the TP sends the request message *m*_req_, signature *σ*_*C*_3__, and cipher message *C*_3_ to the LO.

**Step 4**

Once receiving the message (*m*_req_, *σ*_3_, *C*_3_), the LO checks the signature validity as follows:

(35) $$\begin{array}{c} s_{\text{TP}}^{e_{\text{PKG}}}\;\underline{\underline{?}}\;{\text{ID}}_{\text{TP}}\cdot t_{\text{TP}}^{H(t_{\text{TP}},C_{3})}\mathrm{mod}N_{\text{PKG}}. \end{array}$$

The LO uses its private key *d*_LO_ to decrypt the cipher text as follows:

(36) $$\begin{array}{c} \left(w_{1_{\text{TP}}},w_{2_{\text{TP}}},\ldots ,w_{j_{\text{TP}}},\text{PL}\right)=D\left(K_{\text{TP-LO}},\left(C_{3}\right)\right). \end{array}$$

According to the purchase list PL, the LO uses its private key *d*_LO_ to compute the partial multisignatures *w*_*j*_LO__of all players as follows:

(37) $$\begin{array}{c} w_{j_{\text{LO}}}=d_{\text{LO}}\cdot r_{\text{LO}}^{H(T,\beta_{j},\text{Num})}\mathrm{mod}N_{\text{PKG}},\;\text{for}\;j=1,2,\ldots ,n \end{array}$$

and then LO uses the partial multisignatures of *w*_*j*_TP__and *w*_*j*_LO__to compute

(38) $$\begin{array}{c} W_{j}=w_{j_{\text{TP}}}\cdot w_{j_{\text{LO}}}\;\mathrm{mod}N_{\text{PKG}},\;\text{for}\;j=1,2,\ldots ,n. \end{array}$$

We denote the lottery lottery_*j*_ as

(39) $$\begin{array}{c} {\text{lottery}}_{j}=\left(W_{j},T\right),\;\text{for}\;j=1,2,\ldots ,n. \end{array}$$

### 3.6. The Lottery Issue Phase

**Figure 4:**
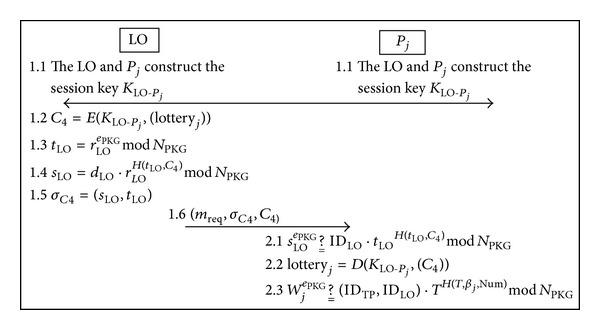
Overview of the lottery issue phase.

Upon receiving the purchase message, the LO issues the lotteries to all players (including the joint purchase and individual purchase) and then the players can apply the multisignature to verify the validity of the lottery. The lottery issue phase is illustrated in Figure 4.

**Step 1**

The LO and *P*_*j*_ construct the session key *K*_LO-*P*_*j*__ and then encrypt the lottery_*j*_ as follows:

(40) $$\begin{array}{c} C_{4}=E\left(K_{\text{LO-}P_{j}},\left({\text{lottery}}_{j}\right)\right). \end{array}$$

Next, the LO selects random number *r*_LO_ to compute

(41) $$\begin{array}{c} t_{\text{LO}}=r_{\text{LO}}^{e_{\text{PKG}}}\mathrm{mod}N_{\text{PKG}} \end{array}$$

and uses its private key *d*_LO_ to compute the partial signature as follows:

(42) $$\begin{array}{c} s_{\text{LO}}=d_{\text{LO}}\cdot r_{\text{LO}}^{H(t_{\text{LO}},C_{4})}\mathrm{mod}N_{\text{PKG}}. \end{array}$$

Here, we denote the signature of *C*_4_ as follows:

(43) $$\begin{array}{c} \sigma_{C_{4}}=\left(s_{\text{LO}},t_{\text{LO}}\right). \end{array}$$

Afterward, the LO sends the request message *m*_req_, signature *σ*_*C*_4__, and ciphertext *C*_4_ to *P*_*j*_.

**Step 2**

When receiving the message (*m*_req_, *σ*_4_, *C*_4_), *P*_*j*_ checks the validity of signature as follows:

(44) $$\begin{array}{c} s_{\text{LO}}^{e_{\text{PKG}}}\;\underline{\underline{?}}\;{\text{ID}}_{\text{LO}}\cdot t_{\text{LO}}^{H(t_{\text{LO}},C_{4})}\mathrm{mod}N_{\text{PKG}} \end{array}$$

and then uses the session key *K*_LO-*P*_*j*__ to decrypt ciphertext *C*_4_ as follows:

(45) $$\begin{array}{c} {\text{lottery}}_{j}=D\left(K_{\text{LO-}P_{j}},\left(C_{4}\right)\right). \end{array}$$

Finally, *P*_*j*_ checks the validity of signature as follows:

(46) $$\begin{array}{c} W_{j}^{e_{\text{PKG}}}\;\underline{\underline{?}}\;\left({\text{ID}}_{\text{TP}},{\text{ID}}_{\text{LO}}\right)\cdot T^{H(T,\beta_{j},\text{Num})}\mathrm{mod}N_{\text{PKG}}. \end{array}$$

### 3.7. The Winning Numbers Generation and Verification Phase

**Figure 5:**
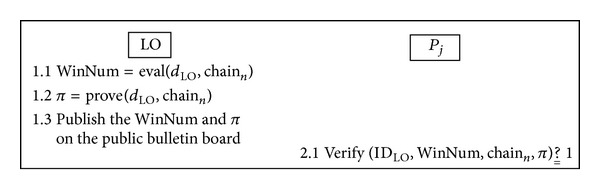
Overview of the winning numbers generation and verification phase.

After the lottery purchase deadline, the LO uses the function of winning numbers generation with the value of final hash chain to generate the winning numbers and then publishes it on the bulletin board. The overview of the winning numbers generation and verification phase is illustrated in Figure 5.

Simultaneously, if the players question whether or not the LO is honest, they can use the public verification function to verify the correctness of the winning numbers.

**Step 1**

The LO uses its private key *d*_LO_ and the value of final hash chain chain_*n*_ to calculate

(47) $$\begin{array}{c} \text{WinNum}=\text{Eval}\left(d_{\text{LO}},{\text{chain}}_{n}\right)\\ \pi =\text{Prove}\left(d_{\text{LO}},{\text{chain}}_{n}\right). \end{array}$$

Finally, the LO publishes the WinNum and *π* on the bulletin board.

**Step 2**

After the winning numbers are published, any player can checkthecorrectness of the winning numbers via the public verification function as follows:

(48) $$\begin{array}{c} \text{Verify}\left({\text{ID}}_{\text{LO}},\text{WinNum},{\text{chain}}_{n},\pi \right)\;\underline{\underline{?}}\;1. \end{array}$$

### 3.8. The Claim Prize Phase

**Figure 6:**
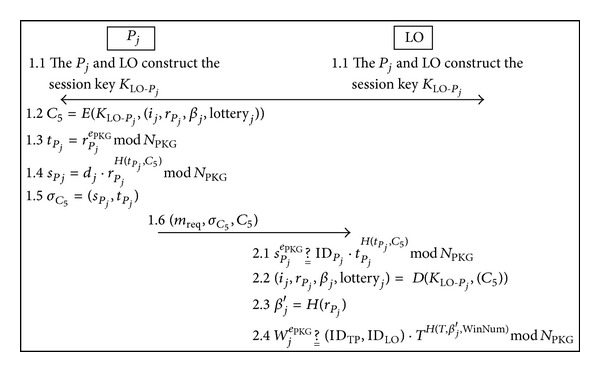
Overview of the claim prize phase.

After the winning numbers are published, the winner of *j*th player can submit his/her winning lottery and the random number to claim the prize. Simultaneously, the LO publishes the winning lotteries, random number of winning player selected, and identity digest of winners on the bulletin board. If the other players suspect the legality of winning lottery, they can use the public verification function to verify it. The overview of the claim prize phase is illustrated in Figure 6.

**Step 1**

The *P*_*j*_ and LO construct the session key *K*_LO-*P*_*j*__. In order to claim the prize, the winning player presents the important evidences (*i*_*j*_, *r*_*P*_*j*__, *β*_*j*_, lottery_*j*_) to prove his/her identity and then uses the session key *K*_LO-*P*_*j*__ to encrypt that evidence as follows:

(49) $$\begin{array}{c} C_{5}=E\left(K_{\text{LO-}P_{j}},\left(i_{j},r_{P_{j}},\beta_{j},{\text{lottery}}_{j}\right)\right). \end{array}$$

Next, the *P*_*j*_ computes as

(50) $$\begin{array}{c} t_{P_{j}}=r_{P_{j}}^{e_{\text{PKG}}}\mathrm{mod}N_{\text{PKG}} \end{array}$$

and uses its private key *d*_*j*_ to compute the partial signature as follows:

(51) $$\begin{array}{c} s_{P_{j}}=d_{j}\cdot r_{P_{j}}^{H(t_{P_{j}},C_{5})}\mathrm{mod}N_{\text{PKG}}. \end{array}$$

Here, we denote the signature of *C*_5_ as follows:

(52) $$\begin{array}{c} \sigma_{C_{5}}=\left(s_{P_{j}},t_{P_{j}}\right). \end{array}$$

Afterward, *P*_*j*_ sends the request message *m*_req_, signature *σ*_*C*_5__, and ciphertext *C*_5_ to LO.

**Step 2**

Once receiving message (*m*_req_, *σ*_5_, *C*_5_), the LO checks the signature validity as follows:

(53) $$\begin{array}{c} s_{P_{j}}^{e_{\text{PKG}}}\;\underline{\underline{?}}\;{\text{ID}}_{P_{j}}\cdot t_{P_{j}}^{H(t_{P_{j}},C_{5})}\mathrm{mod}N_{\text{PKG}} \end{array}$$

and then uses the session key *K*_LO-*P*_*j*__ to decrypt the cipher message *C*_5_ as follows:

(54) $$\begin{array}{c} \left(i_{j},r_{P_{j}},\beta_{j},{\text{lottery}}_{j}\right)=D\left(K_{\text{LO-}P_{j}},\left(C_{5}\right)\right). \end{array}$$

If (53) holds, and then computes *β*_*j*_′ as follows

(55) $$\begin{array}{c} \beta_{j}^{\prime }=H\left(r_{P_{j}}\right). \end{array}$$

Finally, the LO uses the multisignature to verify the correctness of winning lottery as

(56) $$\begin{array}{c} W_{j}^{e_{\text{PKG}}}\;\underline{\underline{?}}\;\left({\text{ID}}_{\text{TP}},{\text{ID}}_{\text{LO}}\right)\cdot T^{H(T,\beta_{j}^{\prime },\text{WinNum})}\mathrm{mod}N_{\text{PKG}}. \end{array}$$

## 4. Analysis

Here, we use many kinds of scenarios to analyze the proposed joint electronic lottery scheme and to verify whether or not it achieves the requirements. In order to simplify the explanation, suppose there exists an intruder* Eve* in the network system and she is capable of eavesdropping communications between the TP, LO, and players.

### 4.1. Public Verification

All the valid lotteries and the winning numbers must be verified via a verifiable random function.

*Scenario 1*. Suppose that any player suspects the correctness of winning numbers.

**Proof**

The one suspecting can use (48) to verify the correctness of the winning numbers by (48).

*Scenario 2*. Suppose that any player suspects the correctness of winning lotteries.

**Proof**

The one suspecting can use the related parameters (including random number of the winning player selected *r*_*P*_*j*__, the winning numbers WinNum, and the winning lottery (*W*_*j*_, *T*)) and (56) to verify the correctness of winning lotteries. The verification equation is as in (56), where *β*_*j*_′ = *H*(*r*_*P*_*j*__).

The derivation of the verification is shown as follows:

(57) (IDTP·IDLO)·TH(T,βj′,WinNum)mod⁡NPKG =(dTPePKG·dLOePKG)·(tTP·tLO)H(T,βj′,WinNum)mod⁡NPKG =(dTPePKG·dLOePKG)·(rTPePKG·rLOePKG)H(T,βj′,WinNum)mod⁡NPKG =(dTPePKG·dLOePKG)·(rTPePKG·H(T,βj′,WinNum)  · rLOePKG·H(T,βj′,WinNum))mod⁡NPKG =((dTP·dLO)·(rTPH(T,βj′,WinNum)·rLOH(T,βj′,WinNum)))ePKG mod⁡ NPKG =((dTP·rTPH(T,βj′,WinNum))·(dLO·rLOH(T,βj′,WinNum)))ePKG mod⁡ NPKG =(wjTP·wjLO)ePKGmod⁡NPKG =WjePKGmod⁡ NPKG.

Because the multisignature of the winning numbers is valid, the winning lottery is correct.

### 4.2. Fairness

No one can predict the winning result before the LO publishes the winning numbers.

*Scenario 3*. If a player wants to predict or bias the winning result, he or she will fail.

**Proof**

Since each purchasing behavior is random and occasional, the final value of hash chain chain_*n*_ is contributed by all of the lotteries. Hence, no one can learn the final value of the hash chain chain_*n*_.

### 4.3. Security

No one can forge winning lotteries or impersonate lottery winners to claim their prize. 

*Scenario 4*. If* Eve* tries to forge a winning lottery to claim the prize, she will fail.

**Proof**

In reviewing the purchase phase, the TP and LO used their private keys *d*_TP_ and *d*_LO_ to sign the lotteries. On the other hand, if* Eve* wants to fake the winning lottery, she must forge their private keys, respectively. In fact, she must solve the factorization problem in RSA cryptosystems [19].

*Scenario 5*. If* Eve* tries to forge a winning player, she will fail.

**Proof**

In the prize claim phase, the lottery winner must submit his/her digest *i*_*j*_, random number *r*_*P*_*j*__ and *β*_*j*_ (where *β*_*j*_ = *H*(*r*_*P*_*j*__)) to proof his/her identity. If* Eve* uses the fake random number *r*_*P*_*j*__′ to claim the prize, then LO can perceive the attempt via the following equation:

(58) $$\begin{array}{c} \beta_{j}\;\underline{\underline{?}}\;H\left(r_{P_{j}}^{\prime }\right). \end{array}$$

On the other hand, if* Eve* wants to impersonate a winning player, she must find the *r*_*P*_*j*__. In fact, based on the secure one way hash function, it is computationally infeasible to obtain *r*_*P*_*j*__ from *β*_*j*_.

### 4.4. Correctness

The players can verify the public information via the bulletin board by themselves.

*Scenario 6*. The one suspecting questions

1. the correctness of the player who bet numbers number_*j*_,
2. the correctness of the value of final hash chain chain_*n*_,
3. the correctness of popular numbers Num.

**Proof**

The one suspecting can use the published bulletin board information to verify the number_*j*_, Num, and chain_*n*_ as

(1) the players can check whether the bet numbers are equal to the public information number_*j*_;
(2) they can recalculate all bet numbers of players to determine whether the popular numbers Num is equal to the recalculated value;
(3) finally, the players can verify the validity of the value of final hash chain chain_*n*_ by using the public function hash chain as follows:
  (59) Initial  condition  chain0=0chain1=H(chain0,β1)chain2=H(chain1,β2)⋮chainn=H(chainn−1,βn),

where *n* is the number of the sold lottery tickets so far.

### 4.5. Anonymity

Including the TP and LO, no one can identify the player from the lottery.

*Scenario 7*. If* Eve* tries to distinguish between messages digest *i*_*j*_ and real identity of player, she will fail.

**Proof**

In the registration phase, the players submit their personal information to the PKG and then PKG generates a message digest with personal information as *i*_*j*_ = *H* (players' personal information).

The message digest is a well-known cryptographic assumption: the secure one way hash function has properties such that given a message *m*, it is easy to compute *H*(*m*). On the other hand, it is computationally infeasible to obtain *m* from *H*(*m*). And given *H*(*m*), it is infeasible to find *m*′ to let *H*(*m*) = *H*(*m*′). Hence* Eve* cannot find the real identity of the player from *i*_*j*_.

### 4.6. Convenience

Players are able to purchase lottery tickets if they can access the Internet. Clearly, the proposed joint e-lottery mechanism can achieve this requirement as indicated in the players betting for lottery numbers phase.

### 4.7. Without Preregistration

Players need not register at any lottery organizations in advance. In our scheme, the players need not register at any lottery organizations except for the PKG. In fact, if the players want to join other ID-based applications, the players still need to register to PKG for any PKI applications.

### 4.8. No Online Trusted Third Party

The proposed joint e-lottery mechanism does not require an online TTP.

In our scheme, no online TTP is used to participate in all of the transaction scenarios. Therefore, this requirement is completed in our scheme.

### 4.9. Participants' Legality

The scenario of the proposed joint e-lottery mechanism should ensure participants' legality.

*Scenario 8*. Suppose that players suspect the legality of the TP and LO.

**Proof**

In the lottery issuing phase, upon the players receiving lotteries from the LO, players can use the multisignature of lotteries to confirm the legality of TP and LO by (46).

If the equation holds, the participants' legality can be authenticated.

That is, only the legitimate private key is able to sign the valid signature. From another viewpoint, the PKG uses its private key *d*_PKG_ to generate *d*_LO_ in the registration phase; if anyone attempts to forge *d*_LO_ he/she must solve the RSA public-key cryptosystem to acquire the private key*d*_PKG_. In fact, it is an integer factorization problem [19].

*Scenario 9*. Suppose that the players, TP, or LO suspect the legality of player.

**Proof**

Anyonesuspecting can authenticate the player's legality by verifying the signature *σ*_*i*_*j*__ = (*s*_*P*_*j*__, *t*_*P*_*j*__) by (9).

If the equation holds, the *j*th player's legality can be authenticated; the derivation of the verification is shown as follows:

(60) ij·tPjH(tPj,ij)mod⁡NPKG =djePKG·tPjH(tPj,ij)mod⁡NPKG =djePKG·(rPjePKG)H(tPj,ij)mod⁡NPKG =(dj·rPj)ePKG·H(tPj,ij)mod⁡NPKG =sPjePKGmod⁡NPKG.

From the above derivation of the verification, only the legitimate private key *d*_*j*_ is able to sign the valid signature. On the other hand, the player is only able to sign the valid signature if he/she registers with the PKG as a legal participant and acquires the private key *d*_*j*_.

### 4.10. Support Joint and Individual E-Lottery Service

The protocol can support joint and individual e-lottery service, respectively. In our proposed scheme, we propose two purchase models to satisfy the requirements. Hence, two purchase models have the same rights and protections making our proposed scheme more practical and attractive.

### 4.11. Discussions

Our scheme focuses on proposing a secure and fair joint e-lottery, despite requiring more communication, more data transfer, and a higher computational complexity. We compare the functional properties between related works and ours in Table 2.

Table 2 shows that our scheme achieves the two new functional properties in comparison with related works [4–6]: participant's legality and supporting joint and individual e-lottery service.

In addition, we compare mechanisms with the existed lottery websites [8, 9] and ours in Table 3. Basically, [8, 9] only support a lottery agent. So, the player should register with the TP; this differs from ours.

Table 3 shows that our scheme adopted the ID-based multisignature to verify the legality of all participants while existing lottery websites lack effective mechanisms to achieve this requirement. On the other hand, the existing websites do not have remedial measures to prevent malicious behaviors by the lottery agent or players; for instance, the lottery agent refuses to give out the prize, a malicious player forges a picture to claim a prize, or the purchased lottery of a player is lost when the lottery agent's database crashes. Our scheme uses the ID-based multisignature to provide nonrepudiation evidence to prevent the above situations.

## 5. Conclusions

In this paper, we present a novel joint e-lottery protocol using the multisignature and verifiable random function. Having been proved, the new mechanism can achieve the requirements of general electronic lotteries. The players can increase the probability of winning prizes by using the proposed secure and fair joint e-lottery scheme. Notably, anyone can verify the correctness of winning lotteries and participants' legality simultaneously by verifying the multisignature; this functionality increases the convenience and security when a new participant joins the system. In the future, we are going to integrate the cash flow concept into our system.

## Figures and Tables

**Table 1:** The information of bulletin board.

Initial condition			
chain_0_ = 0			
chain_1_ = *H*(chain_0_, β_1_)			
chain_2_ = *H*(chain_1_, β_2_)			
⋮			
chain_*j*_ = *H*(chain_*j*−1_, β_*j*_)			
Identity information	Hash chain value	Hash value of the random number *r*_P_*j*__	Selected favorite number

(*i*_1_, σ_*i*_1__)	chain_1_	β_1_	number_1_
(*i*_2_, σ_*i*_2__)	chain_2_	β_2_	number_2_
⋮	⋮	⋮	⋮
(*i*_*j*_, σ_*i*_*j*__)	chain_*j*_	β_*j*_	number_*j*_

**Table 2:** Comparisons between related works and ours.

	Chow et al.'s [4]	Lee and Chang's [5]	Lee et al.'s [6]	Ours
Security	Yes	Yes	Yes	Yes
Correctness	Yes	Yes	Yes	Yes
Anonymity	Yes	Yes	Yes	Yes
Random generation	Yes	Yes	Yes	Yes
Public verification	Yes	Yes	Yes	Yes
Fairness	Yes	Yes	Yes	Yes
Convenience	Yes	Yes	Yes	Yes
No online trusted third party	Yes	Yes	Yes	Yes
No pre-registration required	Yes	Yes	Yes	Yes
Participants legality	No	No	No	Yes
Support joint and individual e-lottery service	Support individual only	Support individual only	Support individual only	Yes

**Table 3:** Comparisons with the existing e-lottery websites.

	E-Lottery Syndicates [8]	Myleto [9]	Ours
Support joint and individual e-lottery service	Support individual only	Support joint only	Support joint and individual
Player should register with the TP	Yes	Yes	No
Allows players to verify legality of lottery agent	Absence of verification mechanisms	Absence of verification mechanisms	Adopts ID-based multi-signature
Allows players and lottery agent to verify the legality of other players	Absence of verification mechanisms	Absence of verification mechanisms	Adopts ID-based signature
The lottery agent refuses to give out the prize	No remedial measures	No remedial measures	Players hold the TP's signature to arbitral request
If a malicious player forges a picture to claim prize	Not easy to identify the legal lottery	Not easy to identify the legal lottery	Prompt identification by digital signature
Non-repudiation evidence	Depend on the scanned copy of the lottery shown on the screen	Depend on the scanned copy of the lottery shown on the screen	PKI digital signature
